# Metallothionein: A Comprehensive Review of Its Classification, Structure, Biological Functions, and Applications

**DOI:** 10.3390/antiox13070825

**Published:** 2024-07-09

**Authors:** Ruoqiu Yang, Dumila Roshani, Boya Gao, Pinglan Li, Nan Shang

**Affiliations:** 1 Key Laboratory of Precision Nutrition and Food Quality, College of Food Science and Nutritional Engineering, China Agricultural University, No, 17 Qinghua East Road, Haidian District, Beijing 100083, China; yangruoqiu@cau.edu.cn (R.Y.); s20213061034@cau.edu.cn (B.G.); 2 College of Engineering, China Agricultural University, No, 17 Qinghua East Road, Haidian District, Beijing 100083, China; dumila@cau.edu.cn

**Keywords:** metallothionein, antioxidant, heavy metal detoxification, neuroprotective, anticancer, anti-inflammatory

## Abstract

Metallothionein is a cysteine-rich protein with a high metal content that is widely found in nature. In addition to heavy metal detoxification, metallothionein is well known as a potent antioxidant. The high sulfhydryl content of metallothionein confers excellent antioxidant activity, enabling it to effectively scavenge free radicals and mitigate oxidative stress damage. In addition, metallothionein can play a neuroprotective role by alleviating oxidative damage in nerve cells, have an anticancer effect by enhancing the ability of normal cells to resist unfavorable conditions through its antioxidant function, and reduce inflammation by scavenging reactive oxygen species. Due to its diverse biological functions, metallothionein has a broad potential for application in alleviating environmental heavy metal pollution, predicting and diagnosing diseases, and developing skin care products and health foods. This review summarizes the recent advances in the classification, structure, biological functions, and applications of metallothionein, focusing on its powerful antioxidant effects and related functions.

## 1. Introduction

Metal ions play an important role in cellular energy transfer, signaling, and other life activities; however, excessive concentrations can be toxic to cells. Cells have evolved two metal resistance mechanisms: metal membrane transport and intracellular chelation, and metallothionein is a chelator that responds to stimuli.

Metallothionein is a class of low-molecular-weight, cysteine-rich, high-metal-content proteins that was first discovered and isolated from equine kidneys by Margoshes and Valle in 1957 [1,2]. Metallothionein is widely distributed in nature and has been found in vertebrates, invertebrates, plants, and microbes [3]. Since the day of its discovery, metallothionein’s distinct structure has drawn attention. Its structure gives it specificity, stability, and dynamic variability while also greatly enhancing its antioxidant and metal ion binding capacities [4]. Metallothionein is capable of chelating metal ions via sulfhydryl groups to exert metal detoxification [5]. The high sulfhydryl content of metallothionein enables it to effectively scavenge various free radicals, such as hydroxyl radicals and oxygen radicals, and the reaction rate constant with hydroxyl radicals reaches 300 times that of glutathione [6]. Metallothionein, by virtue of its metal ion binding and antioxidant capacity, is able to reduce the damage caused by metal ions and oxidative stress on nerve cells [7,8]. In addition, as an antioxidant, it can protect normal cells from external damage and reduce the risk of cancer [9]. When an inflammatory response occurs, metallothionein is able to scavenge reactive oxygen species to exert an anti-inflammatory effect and can also promote tissue repair and regeneration to a certain extent [10]. In recent years, many additional studies on the biological functions of metallothionein have appeared, but there has been no recent comprehensive summary of its functions. Metallothionein, as a highly effective antioxidant, can play an important role in a variety of physiological and biochemical processes. A comprehensive understanding of the function of metallothionein is important for its maximum exploitation. The aim of this review is to introduce the classification, structure, biological function, and current application of metallothionein, with a focus on summarizing the various biological functions of metallothionein with a view to providing reference for its further development and utilization.

## 2. The Classification and Structure of Metallothionein

### 2.1. Classification

**Figure 1:**
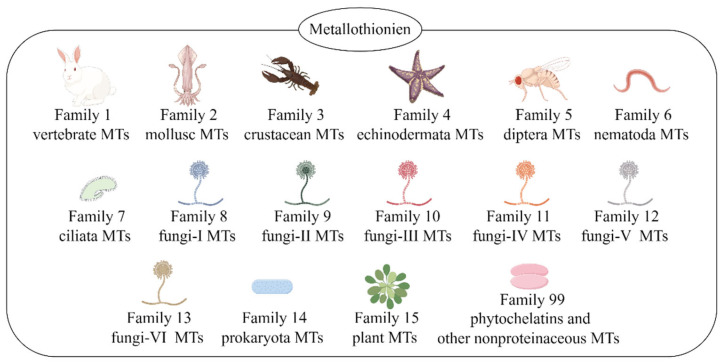
Classification of metallothionein. This figure was drawn by Figdraw (www.figdraw.com accessed on 27 June 2024).

Binz et al. proposed a new classification system to better distinguish between different species of metallothionein. They defined metallothionein as a superfamily, under which it is divided into various families based on evolutionary correlations. There are 15 families in the metallothionein superfamily, including vertebrate MTs, mollusc MTs, crustacean MTs, prokaryotic MTs, plant MTs, etc. (Figure 1) [11]. The human MT family is divided into four classes, MT1 to MT4, encoded by 11 active genes (*MT1A*, *MT1B*, *MT1E*, *MT1F*, *MT1G*, *MT1H*, *MT1M*, *MT1X*, *MT2A*, *MT3*, and *MT4*) located in the chromosome 16 cluster [12]. MT1 and MT2 are expressed in most organs and tissues; MT3 is mainly expressed in the brain, whereas MT4 is expressed in stratified squamous epithelial cells [13,14,15].

### 2.2. Structure

**Figure 2:**
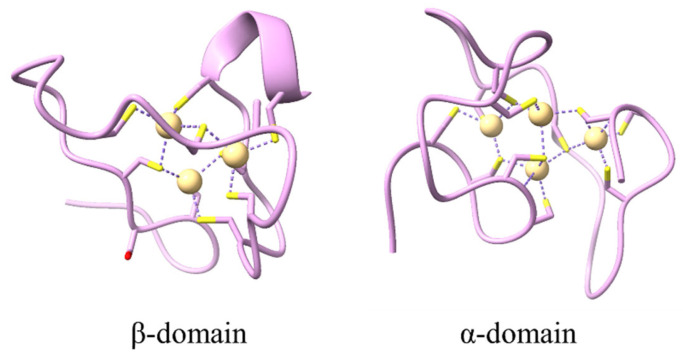
Biological functions of metallothionein. The NMR structure of the α-domain and β-domain of rat MT-2. The models were generated with UCSF ChimeraX (https://www.rbvi.ucsf.edu/chimerax accessed on 27 June 2024) using RCSB PDB (https://www.rcsb.org/ accessed on 27 June 2024) coordinates of 1MRT and 2MRT. The α-domain has four metal ion binding sites, and the β-domain has three metal ion binding sites.

Apoproteins do not form any typical secondary structures when they do not bind to metal ions [16]. Metallothionein acquires its structure only after binding to metals, and its tertiary structure depends on the nature and quantity of the metal ions [17]. Metallothionein is linked to metals mainly through the thiol group in cysteine residues, resulting in the formation of metallothionein clusters with a strong binding capacity for metal ions such as Cu^2+^ and Zn^2+^ [18,19]. Mammalian metallothionein has a relatively unique 3D structure, containing two independent structural domains that combine to form a dumbbell-like structure [20]. The N-terminus is the β structural domain, and the C-terminus is the α structural domain [21]. This structure is also regarded as the model for metallothionein (Figure 2) [22].

## 3. Biological Functions of Metallothionein

**Figure 3:**
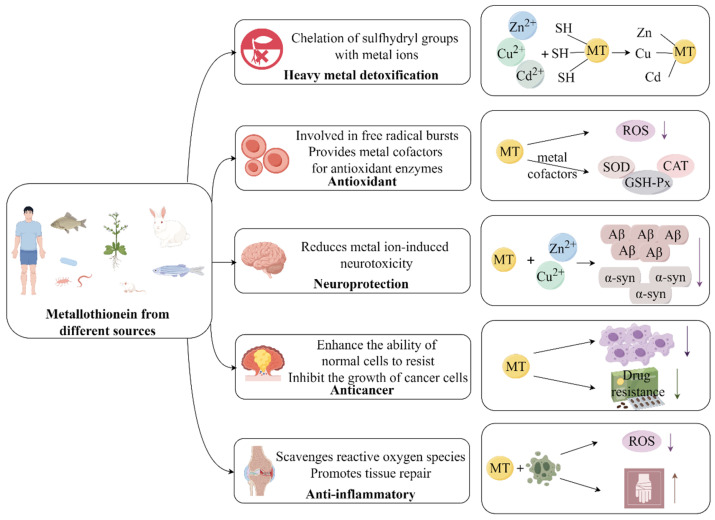
Biological functions of metallothionein. This figure was drawn by Figdraw (www.figdraw.com accessed on 27 June 2024).

Because of its high sulfhydryl content and potent metal ion binding capacity, metallothionein is beneficial in a variety of biological processes [4]. As was already mentioned, several studies have demonstrated that metallothionein has different functions, including the detoxification of heavy metals, antioxidant activity, neuroprotection, anticancer activity, and anti-inflammatory effects (Figure 3).

### 3.1. Detoxification of Heavy Metals

The ability to chelate metal ions is attributed to the abundance of cysteine residues with sulfhydryls present in metallothionein [5]. Table 1 lists studies related to the detoxification of different metal ions by metallothionein. The chelation of metallothionein with metal ions is one of the most crucial pathways for metal detoxification in living cells [23]. Its degradation occurs in the liver after binding to metal ions [24]. When ZnMT is degraded, zinc is rapidly released from it, which continues to induce the production of new metallothionein. CuMT, on the other hand, undergoes oxidation to form insoluble polymers that accumulate in lysosomes and eventually enter the bile [25].

Cadmium poisoning can cause damage to organs such as the liver and kidneys, trigger osteoporosis, increase the risk of cancer, affect the immune system, and trigger metabolic disorders [26]. Metallothionein was initially discovered in cadmium-rich environments and found to be bound to cadmium [1]. It has been shown that metallothionein plays an important role in cadmium detoxification. Studies have demonstrated that *E. coli* cells expressing metallothionein exhibit improved growth compared with those without metallothionein expression when cultured under different cadmium concentrations, indicating the significance of metallothionein cadmium detoxification [27]. The introduction of surface-engineered metallothionein bacteria into rice-growing soil can improve plant height and spike length, lower the cadmium concentration in cadmium-contaminated rice hulls, roots, shoots, and other parts of the rice, and significantly reduce cadmium toxicity [28]. Expression of the *PtMT2b* gene from *Populus trichocarpa* using *S. cerevisiae* effectively increased the yeast’s tolerance to Cd^2+^ until the Cd^2+^ concentration reached 50 μM, at which point its growth was completely inhibited [29]. Expression of IaMT in *E. coli* and determination of cell growth and Cd^2+^ accumulation under a Cd^2+^ environment revealed that IaMT was able to both enhance the tolerance of *E. coli* to Cd^2+^ and also increase the amount of intracellular Cd^2+^ accumulation and improve *E. coli*’s tolerance to Cd^2+^ [30]. A sub-acute concentration of CdCl_2_ solution was injected into grass carp, and MT was administered as an antidote four days later. It was discovered that the grass carp’s blood and kidney Cd levels significantly decreased after receiving an MT injection. Simultaneously, MT significantly decreased blood apoptosis, mitigated the hemoglobin decrease brought on by a Cd injection, and assisted in lessening the immune system’s reaction to Cd [31].

Furthermore, metallothionein exhibits detoxifying effects against various other heavy metal elements. Metallothionein can lessen the harmful effects of As^3+^ on cells, as well as the disruption of phospholipid metabolism and damaged cell membranes caused by As^3+^. Metallothionein chelates As^3+^ by forming a complex with As^3+^ and scavenges ROS, thus alleviating the toxic effects of As^3+^ [32]. ShMT, a metallothionein in freshwater crabs expressed in *E. coli*, has a strong binding ability to Zn, Cu, and Cd ions, with the order of affinity being Cu > Cd > Zn [33]. The chelating action of metallothionein on metal ions led to a significant increase in Zn^2+^ and Cd^2+^ accumulation in tobacco when the human metallothionein *HsMT1L* gene was expressed in tobacco, improving tolerance to Zn^2+^ and Cd^2+^. *HsMT1L* is capable of removing heavy metal ions and plays a role in protecting plants from heavy metal toxicity [34].

**Table 1:** Studies on the role of metallothionein in heavy metal detoxification.

Metallothionein Source	Target of Action	Type of Metal Ion	Effect	Reference
*Anabaena* PCC 7120 NmtA	*E. coli* cells	Cd^2+^	NmtA-expressing *E. coli* exhibits better growth at certain cadmium concentrations	[27]
Metallothionein expressed by *Alishewanella* sp. WH16-1-MT	Rice	Cd^2+^	Increased plant height, spike length, and thousand-grain weight of rice, resulting in a significant reduction in Cd^2+^ content in brown rice, rice husk, roots, and shoots	[28]
S. cerevisiae expresses the PtMT2b gene from Populus *trichocarpa*	*S. cerevisiae*	Cd^2+^	Enhanced Cd^2+^ tolerance in *S. cerevisiae*	[29]
*Ipomoea aquatica* metallothionein IaMT expressed in *E. coli*	*E. coli*	Cd^2+^	Increased tolerance to and accumulation of Cd^2+^ in *E. coli*	[30]
Rabbit liver MT-2	Grass carp	Cd^2+^	Reduced cadmium levels in kidneys and blood, attenuating organ damage	[31]
Commercial production of metallothionein	PC12 cells	As^3+^	Reduced As^3+^-induced metabolic disturbances and inhibited ROS accumulation	[32]
ShMT expressed in *E. coli*	Metal ions in solution	Zn^2+^, Cu^2+^ and Cd^2+^	SnMT has a strong ability to bind Zn, Cu, and Cd metals	[33]
Human metallothionein *HsMT1L* gene expressed in tobacco	tobacco	Zn^2+^ and Cd^2+^	Increased accumulation of Zn^2+^ and Cd^2+^ in tobacco and enhanced tolerance to Zn^2+^ and Cd^2+^	[34]
Metallothionein expressed by the engineered bacterium EcN-MT	Mice	Cd^2+^	Significantly reduced the content of Cd in mouse liver and accelerated the metabolism of Cd	[35]
Metallothionein expressed by *Rhizobium leguminosarum* strains expressing the pea metallothionein gene	CdCl_2_ solution	Cd^2+^	Enhanced Cd tolerance in peas	[36]

### 3.2. Antioxidant Effect

Free radicals are produced during normal human metabolic processes and are also induced by exposure to radiation, ozone, and air pollutants [37]. When there is an imbalance between the production of free radicals and antioxidant defenses, it can lead to oxidative damage to nucleic acids, proteins, and lipids in the body, resulting in oxidative stress, which has an impact on the function of the body’s antioxidant system [38,39]. Oxidative stress triggers a variety of diseases, including inflammatory diseases, neurological disorders, cardiovascular diseases, cancer, and more [37]. Sulfhydryl groups serve as efficient scavengers of free radicals, making them preferred targets for their neutralization [40]. Metallothionein, as one of the major sources of thiols in cells, contains cysteine residues that can reduce oxidative damage by both preventing the generation of ROS and helping to quench it in vivo [40,41,42]. Metallothionein can also provide metal cofactors for some antioxidant enzymes to exert antioxidant effects [43]. Metallothionein exhibits potent antioxidant properties, effectively scavenging various free radicals, such as hydroxyl and superoxide radicals. Its reaction rate constant with hydroxyl radicals is approximately 300 times higher than that of glutathione [6]. In addition, metallothionein has about 50-fold higher antioxidant activity against oxidative DNA damage and about 10-fold higher antioxidant activity against lipid peroxidation than glutathione [44]. MTF-1 and Nrf2 would regulate metallothionein expression by activating the ARE in the promoter region, while Nrf2 and its downstream antioxidant genes may also be regulated by metallothionein [45]. Several studies have confirmed the antioxidant effects of metallothionein (Table 2).

The ability of *E. coli* to express GST-AmMT2 increased the bacteria’s tolerance to H_2_O_2_, which may be explained by the fact that GST-AmMT2 increases CAT activity [46]. When metallothionein knockout mice and normal mice were subjected to intermittent hypoxic conditions, the former showed reduced lung structure damage and a shorter duration of oxidative stress [45]. Furthermore, another experiment using a knocked-out mouse metallothionein gene showed that when PPE was injected into the trachea of mice, almost no ROS was produced in the lungs of normal mice, but a higher amount was produced in the lungs of mice with the metallothionein gene knocked out [47]. The addition of rh-MT-III to oxidatively damaged *Caenorhabditis elegans* nematodes revealed restoration of motility, reduction of malondialdehyde and reactive oxygen species levels, enhancement of the antioxidant defense system, and alleviation of the extent of oxidative damage [48]. A certain degree of improvement in the tolerance of yeast to oxidative stress was observed when the *PdMT2A* gene was expressed, leading to an increase in the tolerance of yeast to H_2_O_2_. Expression of the *PdMT2A* gene in transgenic *Arabidopsis* seedlings produced seedlings that were considerably more SOD-active and more resistant to oxidative stress than normal plants [42]. The effect of MT3 on ROS production was examined when osteoblasts were differentiated in C2C12 cells. The findings demonstrated that when MT3 was overexpressed, ROS production was suppressed, and when MT3 was silenced, ROS production increased [49]. In HT1376 cells, MT2A knockdown raised endogenous ROS levels. In contrast, overexpression of MT2A inhibited H_2_O_2_-induced ROS production in the cells [50]. ROS levels are elevated during the early stages of adipose differentiation. Overexpression of MT3 reduces ROS levels. When MT3 expression was knocked down, the level of ROS was significantly increased [51]. LPS induces oxidative damage in the heart, mainly by increasing the production of superoxide anion radicals and reducing glutathione levels. The presence of metallothionein attenuates the degree of oxidative stress induced by LPS [52]. Overexpression of *GmMT-II* resulted in a significant increase in SOD, CAT, and POD activities in all transgenic *Arabidopsis* lines under high temperature and humidity stress [53]. When *LcMT3* was expressed in *Arabidopsis*, the amount of MDA and ROS decreased and the expression of SOD, CAT, and POD increased [54].

### 3.3. Neuroprotective Effect

Alzheimer’s disease is a type of dementia that is prevalent in the elderly and is characterized by the deposition of amyloid-β peptide (Aβ), forming localized amyloidosis [55]. Aβ plaques are rich in metal ions, such as zinc and copper, which may induce the aggregation of Aβ peptides, leading to Alzheimer’s disease [56]. The main pathological hallmark of Parkinson’s disease is the accumulation of Lewy bodies, whose main protein component is α-synuclein (α-syn) [7]. Increased levels of metal ions trigger the misfolding of α-syn, which causes the formation of Lewy bodies and Parkinson’s disease [57]. Metallothionein has a strong metal ion binding capacity, is able to reduce metal ion-induced neurotoxicity, and thus may play an important role in the treatment of diseases such as Alzheimer’s disease and Parkinson’s disease [7,55].

Prolonged metallothionein action resulted in decreased toxicity of Aβ and α-syn in aged transgenic Caenorhabditis elegans [58]. Aβ_1-42_ leads to increased Zn^2+^ content in dentate granule cells and impairs hippocampus-dependent memory. Dexamethasone injection increases metallothionein expression and maintains Zn^2+^ homeostasis [59]. The formation of α-syn-Cu (II) complexes catalyzes toxic reactions and accelerates neuronal death. The addition of Zn_7_MT-3 removes Cu (II) and effectively inhibits the toxic effects of α-syn-Cu (II) [60].

Numerous other studies have demonstrated the neuroprotective properties of metallothionein in addition to these two disease-related investigations. Several studies on the neuroprotective effects of metallothionein have been listed in Table 3. Paraquat causes brain damage in zebrafish. Treatment with hMT2 has been shown to mitigate the negative effects of paraquat, including decreased lipid peroxidation and dopaminergic neurons [61]. Overexpression of MT3 in astrocytes resulted in lower levels of glutamate in the culture medium compared with control cells, demonstrating that MT3 expression enhances the buffering capacity of astrocytes against glutamate, thereby reducing the neurotoxicity caused by glutamate [62]. Rat spinal motor neurons were shown to benefit neuroprotectively from the addition of zonisamide, which was able to increase the expression of astrocyte metallothionein 2A and lessen oxidative stress-induced astrocyte damage [8]. Wild mice chronically exposed to an active volcano environment had high levels of heavy metal elements in their brains and were detected to have higher levels of MT-2A than normal mice. By chelating heavy metal ions, metallothionein mitigates the damage that volcanic pollutants cause to the central nervous system [63]. Injection of isoproterenol into mice resulted in increased synthesis of metallothionein, which helped to reduce Aβ_1-42_-induced toxicity [64].

### 3.4. Anticancer Effect

As mentioned above, metallothionein has the functions of heavy metal detoxification and reduction of oxidative stress damage, which can enhance the ability of normal cells to resist external unfavorable conditions, thus having an anti-cancer effect [9]. At the same time, metallothionein can inhibit cancer cell growth, migration, and invasion and can induce cell cycle arrest in cancer cells, leading to apoptosis [65]. Studies on the anticancer effects of metallothionein have been listed in Table 4.

Furthermore, metallothionein has the effect of detoxifying heavy metals and reducing oxidative stress damage, which can enhance the adaptability of cells to anticancer drugs and alter cancer cells’ resistance to chemotherapy, both of which have an anticancer effect.

Evaluation of metallothionein expression in feline injection site fibrosarcoma revealed that there was a negative correlation between the tumor grade and the level of inflammation. Cells are protected from oxidative stress damage by metallothionein, and its down-regulation may raise the risk of DNA damage and, consequently, cancer risk [9]. In esophageal squamous cell carcinoma cells, overexpression of MT1M induces apoptosis, reduces cell viability, and inhibits epithelial-mesenchymal transition, thus acting as a tumor suppressor [66]. Cannabidiol was able to exert a therapeutic effect on colorectal cancer, and overexpression of MT1G and MT2A increased the number of dead cells and synergistically enhanced the anticancer effect of cannabidiol [67]. Overexpression of MT1E in hepatocellular carcinoma cells resulted in a significant decrease in both cell viability and cell number. Following MT1E knockdown, there was a notable decrease in the quantity of apoptotic cells [68]. Overexpression of MT1M inhibited the proliferation, migration, and invasion of gastric cancer cells and promoted apoptosis of gastric cancer cells. Meanwhile, MT1M may also play an anti-cancer role by decreasing the stemness of gastric cancer cells and increasing their sensitivity to 5-fluorouracil [69]. MT1G inhibited the proliferation, migration, and invasion of hepatocellular carcinoma cells in both in vivo and in vitro experiments. When overexpressed, it also demonstrated synergistic inhibition with sorafenib. When the expression of MT1G was disrupted, the effect of sorafenib was attenuated, indicating that MT1G was involved in the process of sorafenib action [70]. Overexpression of MT-1 can delay the progression of hepatocellular carcinoma. Decreased expression of MT-1 can also assist in the diagnosis of hepatocellular carcinoma to a certain extent [71]. Overexpression of MT2A inhibits proliferation and migration of colorectal cancer cells as well as growth and metastasis of cancer cells [72].

Metallothionein can also play a role in the treatment of cancer by influencing chemotherapy resistance. There is a problem of resistance to gemcitabine in the treatment of pancreatic ductal adenocarcinoma, and MT1G is able to limit the secretion of activin A and inhibit pancreatic ductal adenocarcinoma cell stemness, thus overcoming the resistance to gemcitabine [73].

In addition, metallothionein has a tendency to chelate with chemotherapy medications, lowering the medications’ toxicity to cancer cells [74]. Malignant pleural mesothelioma can be successfully treated with platinum-based chemotherapy; however, metallothionein has the potential to bind to cisplatin and make it inactive. Knockdown of MT2A expression in malignant pleural mesothelioma cell lines resulted in a significant increase in apoptosis in response to cisplatin. Thus, inhibition of MT2A expression can significantly improve the therapeutic effect of cisplatin [75]. Chemotherapy resistance is a major challenge in the treatment of osteosarcoma. Four chosen osteosarcoma cell lines exhibit increased sensitivity to several chemotherapeutic agents when MT2A is silenced, which promotes the effectiveness of chemotherapeutic agents [76]. Carbon monoxide reduces levels of metallothionein, which reduces drug resistance in ovarian cancer cells. The therapeutic effect of cisplatin can be enhanced by decreasing the expression of metallothionein [77].

**Table 4:** Studies on the anticancer effect of metallothionein.

Metallothionein Type	Type of Cancer	Effect	Reference
Metallothionein I-II	Feline injection site fibrosarcoma	The degree of metallothionein expression negatively correlates with the degree of inflammation and tumor grade	[9]
MT1M	Esophageal squamous cell carcinoma	Overexpression of MT1M altered cell morphology, induced apoptosis, decreased cell viability and epithelial-mesenchymal transition, up-regulated ROS levels, down-regulated SOD1 activity, and phosphorylated the SOD1 downstream pathway PI3K/AKT	[66]
MT1G and MT2A	Colorectal cancer	Synergizing with cannabidiol in the treatment of colorectal cancer	[67]
MT1E	Hepatocellular carcinoma	MT1E inhibits hepatocellular carcinoma cell proliferation, migration, and invasion and induces apoptosis	[68]
MT1M	Gastric cancer	Inhibits proliferation, migration, and invasion of gastric cancer cells, promotes apoptosis, increases chemosensitivity to 5-fluorouracil, and inhibits stem cell production	[69]
MT1G	Hepatocellular carcinoma	Inhibition of proliferation, cloning, migration and invasion of hepatocellular carcinoma cells and mediation of the anticancer effect of sorafenib	[70]
MT-1	Hepatocellular carcinoma	Suppression of MT-1 expression leads to proliferation of hepatocellular carcinoma cells	[71]
MT2A	Colorectal cancer	Overexpression of MT2A suppresses proliferation and migration of colorectal cancer cells	[72]
MT1G	Pancreatic ductal adenocarcinoma	MT1G negatively regulates NF-κB signaling and limits activin A secretion	[73]
MT2A	Malignant pleural mesothelioma	Knockdown of MT2A expression enhances the apoptosis rate of malignant pleural mesothelioma cells under cisplatin effect	[75]
MT2A	Osteosarcoma	MT2A silencing elevates the sensitivity of osteosarcoma cell lines to multiple chemotherapeutic agents	[76]
Nuclear metallothionein	Ovarian cancer	CO treatment attenuates the expression of nuclear metallothionein in cisplatin-resistant ovarian cancer cell lines and enhances the sensitivity of cisplatin-resistant cell lines to cisplatin	[77]

### 3.5. Anti-Inflammatory Effect

Any response that damages the body results in inflammation. Although the inflammatory process is normally self-limiting, intervention is needed to control acute inflammation and return the immune system to homeostasis [78]. The primary mechanism by which metallothionein exerts its anti-inflammatory properties is through its capacity to scavenge reactive oxygen species. Furthermore, it functions as a zinc chaperone, thereby triggering matrix metalloproteinases that facilitate tissue repair and regeneration during inflammatory conditions [10]. Inflammation can activate metallothionein expression through a variety of pathways, such as stimulation of antioxidant-responsive elements in promoter regions, specific metal-responsive elements, activation of the second messenger protein kinase pathway, and so on [79]. Several studies have reported the anti-inflammatory effects of metallothionein (Table 5).

Studies on synovial cells isolated from patients with osteoarthritis revealed that MT-1 significantly reduced the expression of inflammatory cytokines such as TNF-α and IL-6β and exerted anti-inflammatory effects [80]. Overexpression of MT1 in the mouse liver in a CDAHFD mouse model resulted in the downregulation of genes related to inflammation and fibrosis, such as timp-1, coll1, ten-α, and mcp-1. It was demonstrated that MT1 could alleviate liver fibrosis and improve non-alcoholic steatohepatitis to a certain extent [81]. Naringenin was able to reduce the expression of pro-inflammatory cytokines by inducing MT1G expression and exerting an inhibitory effect on NF-κB activation. Pro-inflammatory cytokine expression rises after MT1G is knocked down [78]. Zinc supplementation improved the treatment of colitis in a mouse model of the disease by upregulating the expression of MT1 and MT2, regulating intestinal inflammation in terms of intestinal epithelial integrity, the immune system, metabolic function, and the defense against oxidative stress [82]. When As^3+^ causes an inflammatory response in carp gills, Zn^2+^ can cause metallothionein to be expressed, prevent the NF-κB signaling pathway from being activated, decrease the number of inflammatory factors secreted, and ultimately stop the inflammatory response [83]. MT1 expression was similarly up-regulated during CuL5’s anti-inflammatory effect on microglia, suggesting a potential correlation between MT1 and CuL5’s anti-inflammatory effect [79]. During clearance of gram-negative bacterial infections, the non-canonical inflammasome of mouse caspase-11 is activated, but this process also causes severe inflammatory injury. MT3 expression increases Zn^2+^ levels, inhibits caspase-11 signaling through the TRIF-IRF3-STAT1 axis, and controls inflammation development [84]. Polysaccharides extracted from *Plantago asiatica* L. seeds were able to protect against inflammatory liver injury in mice to a certain extent. This resulted in an increase in metallothionein levels, which may have an anti-inflammatory effect by scavenging excess ROS generated during LPS-induced liver injury [85]. MT-1 levels were elevated in patients with ankylosing spondylitis and positively correlated with ankylosing spondylitis activity and inflammatory response, suggesting that MT-1 may be involved in defense systems against inflammatory processes [86]. An inflammatory response occurs during pre-eclampsia, and supplementation of zinc gluconate to pre-eclamptic rats increased MT levels, which significantly reduced pro-inflammatory cytokine levels and helped to alleviate the inflammatory response [87]. Exosomes from mesenchymal stromal cells were able to up-regulate the expression of MT-2, elevate the transcriptional level of IκBα in macrophages of mice with colitis, and inhibit the activation of NF-κB. This increased macrophage resistance to inflammation, which in turn facilitated the treatment of colitis [88]. Significantly elevated levels of Mt1 and Mt2 in alcoholic hepatitis mice reduce levels of lipid peroxides such as 4-hydroxynonenal and malondialdehyde and decrease the activation of stress kinases [89]. MT-1 and MT-2 inhibit the formation of osteoclasts and prevent osteoporosis and other damage caused by rheumatoid arthritis [90]. The ethyl acetate fraction of *Amomum villosum* var. *xanthioides* alleviates non-alcoholic steatohepatitis by enhancing antioxidant capacity and improving oxidative status through increased MT1 expression [91]. In addition, MT-1 is able to play an immunomodulatory role by regulating the proliferation and differentiation of immune cells and acting as a chemoattractant to regulate cellular infiltration, thus effectively alleviating the inflammatory response [92].

## 4. Applications of Metallothionein

**Figure 4:**
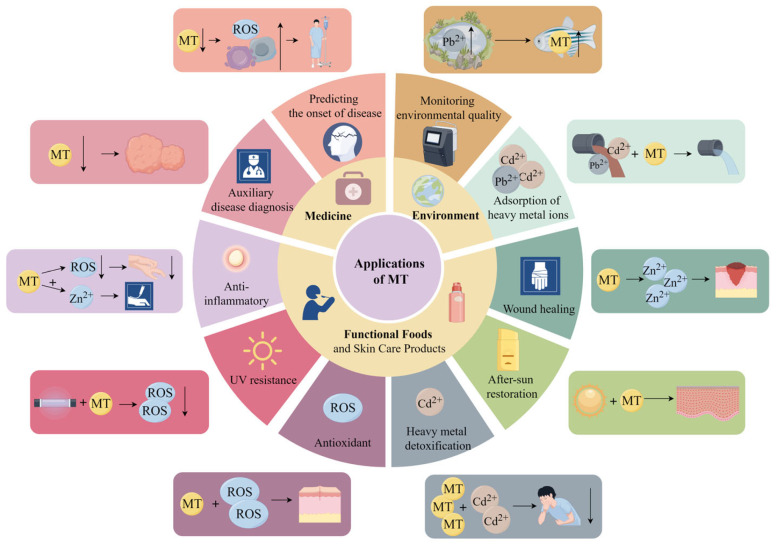
Applications of metallothionein. This figure was drawn by Figdraw (www.figdraw.com accessed on 27 June 2024).

In view of the multiple biological functions of metallothionein, it has a broad application prospect in environment, medicine, food and so on (Figure 4).

### 4.1. Detection and Removal of Heavy Metal Ions from the Environment

Metallothionein can be used as a biomarker for aquatic organisms exposed to heavy metal ions because its gene expression is stimulated by various heavy metal ions and can react at an early stage of environmental pollution [93]. Preparation of fish metallothionein-specific polyclonal antibodies enables the detection of changes in metallothionein levels in the livers of a wide range of fish species, delivering early warning signs before heavy metal ion pollution of aquatic ecosystems occurs [93]. Metallothionein in plankton is also able to reflect the degree of contamination of organisms with heavy metal ions, which can be used in the assessment of environmental quality [94]. Fish levels of metallothionein increased significantly as a result of heavy metal ion pollution in the waters; hence, changes in fish levels of metallothionein can be used to track heavy metal ion pollution in the waters [95].

Metallothionein demonstrates a good ability to bind heavy metal ions. When used in combination with other materials, it can remove heavy metal ions from the environment, thereby reducing the pollution associated with heavy metal ions [96]. Novel biosorbents were constructed using cellulose, metallothionein, and carbohydrate-binding modules, which can effectively adsorb Pb^2+^ and Zn^2+^ from polluted water and play a great role in removing toxic trace elements from water [97]. The synthesized metallothionein SmtA-modified selenium nanoparticles were able to efficiently adsorb and stably remove Cd^2+^ and Pb^2+^ and reduce cadmium and lead residues in wastewater to meet the national wastewater discharge standards [96]. After being genetically modified and co-assembled with magnetic nanoparticles, the metallothionein gene was introduced into E. coli and successfully removed Pb^2+^ and Cd^2+^, with a removal efficiency of >80% [98]. Cloning of MT2A and MT3 into *E. coli* was effective in removing Cr^6+^ from an aqueous solution, and the main functioning groups were the hydroxyl, phosphoryl, and carbonyl groups [99]. Encapsulation of MT3-expressing *E. coli* in the form of calcium alginate bio-beads was able to effectively remove three heavy metals, copper, zinc, and cadmium, from water, with the most significant removal effect on copper [100]. Dissolved metal ions in mine wastewater are difficult to remove. *Escherichia coli* overexpressing MTA were able to efficiently absorb nickel from wastewater, with a seven-fold increase in the ability to bioaccumulate nickel compared with the control [101].

### 4.2. Disease Prediction and Diagnosis in Medicine

In the field of medicine, it is possible to predict and diagnose disease by detecting changes in the content of metallothionein. Metallothionein has various functions, including antioxidants, heavy metal detoxification, and maintenance of metal ion homeostasis. Its content tends to change during the occurrence of diseases to counteract the adverse state of the organism [102].

Genes like MT1E and MT1F can be used as biomarkers to predict the recurrence of hepatocellular carcinoma. Reduced expression of metallothionein may raise the risk of cancer, as evidenced by the down-regulation of these genes in patient samples with hepatocellular carcinoma [103]. MT-1 is able to predict the development of schizophrenia to a certain extent. The risk of schizophrenia is elevated when the level of MT-1 is reduced, probably due to the lack of metallothionein, which leads to an increased risk of oxidative damage in the body, thus raising the risk of schizophrenia [102]. Wilson disease is an inherited disorder of copper metabolism in which the liver and brain are affected by copper toxicity. Since metallothionein can detoxify copper, the expression of metallothionein is increased in the tissues of patients with Wilson disease. Positive metallothionein immunostaining has been demonstrated to be a useful tool in the diagnosis of Wilson disease [104]. The currently available prognostic indicators of hepatocellular carcinoma, such as tumor number and cell differentiation, have many limitations in the treatment of therapy and prognosis. To increase prognostic accuracy, multi-indicator prediction using oxidative stress biomarkers, like MT-3, can be used [105]. When colorectal cancer occurs, the levels of MT1B, MT1F, and MT1G genes are down-regulated, and MT genes may be able to serve as effective markers for the diagnosis of the disease. In addition, high expression levels of the MT1B, MT1H, and MT1L genes are associated with a good prognosis in colorectal cancer patients and can predict the prognosis of the disease [106]. The greater the MT1X expression in patients with clear cell renal cell carcinoma, the greater the likelihood of both highly graded tumors and metastasizing tumors. Therefore, MT1X can predict tumorigenesis to some extent and predict the prognosis of clear cell renal cell carcinoma [107]. Patients with gastric cancer had down-regulated levels of metallothionein expression, and there was a significant positive correlation between MT mRNA and overall survival (OS), first-progression survival (FP), and post-progression survival (PPS). These results have the potential to predict the prognostic status of gastric cancer patients [108]. MT1JP expression was down-regulated in glioma patients. Glioma patients with high MTIJP expression had a longer survival time relative to those with low expression. Thus, the expression of MTIJP can provide some reference for the diagnosis of glioma [109].

### 4.3. Development of Products with Skincare Functions

Metallothionein has many functions, such as anti-inflammatory, oxidative damage relief, anti-ultraviolet radiation, heavy metal detoxification, post-sunburn repair, accelerated wound healing, etc. The development of skincare products or functional foods with metallothionein as the active ingredient will be widely used in the field of skincare.

The expression of metallothionein genes was significantly elevated in dermatitis patients, and MT1X expression was able to reduce the inflammatory response triggered by allergens. At the same time, MTF1 expression can prevent allergen-induced oxidative stress, thus providing a protective effect on the skin [110]. By binding zinc in the cytoplasm, metallothionein can control zinc homeostasis and delay the onset of the hand-foot skin reaction. Zinc ions also protect the skin from oxidative damage by inducing the synthesis of metallothionein, a preferred target of oxidant attack [111]. UV radiation causes damage to the DNA of living organisms and also produces reactive oxygen species, leading to apoptosis and senescence. By stimulating the synthesis of metallothionein, 1,25-dihydroxyvitamin D3 can decrease the formation of sun-damaged cells and improve the skin’s resistance to UV light [112]. After irradiating the skin with sun-simulated UV radiation and applying skin care products containing isoflavonoids, epidermal cell MT expression increased. Metallothionein exerts photoprotective effects mainly by scavenging free radicals [113]. When the back skin of mice lacking metallothionein was exposed to UVB radiation, the number of sun-damaged cells increased significantly, and the skin thickness was significantly higher than in control mice [114]. The skin was repaired after exposure to the sun, and the expression of MT1A was elevated. Metallothionein has a strong antioxidant capacity, and the elevated expression may be related to its involvement in skin cell repair after exposure to the sun [115]. When substances containing cadmium are applied to the skin, cadmium is readily absorbed into the skin and poses a health hazard. Cadmium causes apoptosis in keratin-forming cells, and curcumin is able to protect cells from cadmium toxicity damage by up-regulating the expression of MT2A and binding to cadmium [116]. After skin damage, the newly growing epidermis and skin cells at the wound edge express more metallothionein than does the surrounding normal skin. The increased expression of metallothionein may contribute to the enhancement of the anti-inflammatory activity of zinc and the enhancement of MMP-1 expression to promote the migration of keratinocytes, thus contributing to the healing of skin wounds [117].

## 5. Conclusions

Metallothionein plays a very important role in heavy metal detoxification, antioxidants, neuroprotection, anticancer, and anti-inflammatory activities due to its high metal ion binding capacity and high sulfhydryl content. The diverse biological functions of metallothionein also give it a broad application prospect in the fields of environmental protection, disease diagnosis and treatment, and skin care. However, the structure of metallothionein varies greatly among different species, and the metal ion binding state of metallothionein in vivo cannot be identified, resulting in the function of a particular metallothionein and the mechanism of its functioning being unclear, which needs to be further investigated in the future.

## Figures and Tables

**Table 2:** Studies on the antioxidant effect of metallothionein.

Metallothionein Source	Target of Action	Effect	Reference
GST-AmMT2 expressed in *E. coli*	*E. coli*	Enhance tolerance of *E. coli* to H_2_O_2_	[46]
Self-expressed metallothionein in mice	Mice	Relief of oxidative stress and damage to the lungs	[45]
Self-expressed metallothionein in mice	Mice	Inhibited PPE-induced ROS production in the lungs	[47]
rh-MT-III	*Caenorhabditis elegans*	Reduced levels of malondialdehyde and reactive oxygen species	[48]
Metallothionein 2A gene from date palm expressed by yeast and Arabidopsis thaliana	yeast and *Arabidopsis thaliana*	Improved oxidative stress tolerance	[42]
MT3 expressed by C2C12 cells	C2C12 cells	Reduced oxidative stress during osteoblast differentiation	[49]
MT2A gene expressed by HT1376 cells	HT1376 cells	Inhibited H_2_O_2_-induced ROS production	[50]
MT3 expressed by 3T3-L1 cells	3T3-L1 cells	Reduced levels of ROS at the adipose differentiation stage	[51]
Metallothionein expressed in mouse cardiomyocytes	Mouse cardiomyocytes	Reduction of superoxide anion radical production and glutathione levels	[52]
*GmMT-II* expressed by transgenic *Arabidopsis*	Transgenic *Arabidopsis*	Enhanced activities of SOD, CAT, and POD	[53]
*LcMT3* expressed by transgenic *Arabidopsis* thaliana	Transgenic *Arabidopsis*	Reduced accumulation of malondialdehyde and reactive oxygen species and increased activities of SOD, POD, and CAT	[54]

**Table 3:** Studies on the neuroprotective effect of metallothionein.

Metallothionein Source	Target of Action	Effect	Reference
Metallothionein expressed by aged transgenic *Caenorhabditis elegans*	aged transgenic *Caenorhabditis elegans*	Reduction of Aβ and α-syn toxicity	[58]
Metallothionein expressed by dentate granule cells	Dentate granule cells	Chelating Zn^2+^ and maintaining Zn^2+^ homeostasis	[59]
Zn_7_MT-3	α-syn-Cu(II)	Elimination of α-syn-Cu(II) dopamine oxidase activity and removal of Cu(II) from α-syn-Cu(II)	[60]
Human metallothionein 2 peptide (hMT2)	Zebrafish brain	Increased lipid peroxidation and an increased number of dopaminergic neurons	[61]
MT3 expressed by astrocytes	Astrocyte	Increased expression of glutamate transporter protein and glutamine synthetase	[62]
Metallothionein 2A expressed by astrocytes	Astrocyte	Oxidative stress in spinal motor neurons exerts a neuroprotective effect	[8]
MT-2A expressed in the central nervous system	Central nervous system	Higher MT-2A labeling was observed in the subgranular zone and white matter, in the cytoplasm of some cells in the molecular layer, and in the choroid plexus of the brain	[63]
Metallothionein in the cell layer of dentate granules	Dentate granular cell layer	Maintenance of Zn^2+^ homeostasis and reduction of Aβ_1-42_ toxicity	[64]

**Table 5:** Studies on the anti-inflammatory effect of metallothionein.

Metallothionein Type	Type of Inflammation	Effect	Reference
MT-1	Osteoarthritis	MT-1 inhibits inflammatory cytokine expression in synoviocytes	[80]
MT1	Non-alcoholic steatohepatitis	Attenuates steatosis, improves hepatic aspartate aminotransferase, and downregulates timp-1, coll1, ten-α, and mcp-1	[81]
MT1G	LPS induces inflammation in macrophages	Reduced expression of pro-inflammatory cytokines such as TNF-α, IL-1, and IL-1β	[78]
MT1 and MT2	Colitis	Modulation of intestinal inflammation in terms of intestinal tissue protection, epithelial integrity, immune system regulation, targeted digestion, metabolic function, and counteracting oxidative stress	[82]
MT	As^3+^-induced inflammatory responses	Inhibition of NF-κB signaling pathway activation and reduction of inflammatory factor secretion	[83]
MT1	Neuroinflammation	Upregulation of MT1 expression may correlate with CuL5-mediated anti-inflammatory effect	[79]
MT3	Inflammation caused by activation of non-canonical inflammasome	MT3 expression increased Zn^2+^ levels, inhibited caspase-11 signaling through the TRIF-IRF3-STAT1 axis, and decreased activation of caspase-11 and its downstream targets, caspase-1 and IL-1β	[84]
MT	Inflammatory liver injury	May exert anti-inflammatory effects by scavenging excess ROS	[85]
MT-1	Ankylosing spondylitis activity	Elevated MT-1 levels are positively correlated with ankylosing spondylitis activity, inflammatory response, clinical indicators, and pro-inflammatory cytokines	[86]
MT	Inflammatory response during pre-eclampsia	Decreased pro-inflammatory cytokines such as IL-6 and TNF-α	[87]
MT-2	Colitis	Up-regulation of MT-2 expression increases IκBα transcript levels and inhibits IκBα phosphorylation in macrophages of mice with colitis	[88]
MT1 and MT2	Alcoholic hepatitis	Increassed expression of Mt1 and Mt2 decreased the levels of lipid peroxides such as 4-hydroxynonenal and malondialdehyde and reduced the activation of stress kinases	[89]
MT-1 and MT-2	Rheumatoid arthritis	Inhibits osteoclasts and prevents osteoporosis and other damage caused by rheumatoid arthritis	[90]
MT1	Non-alcoholic steatohepatitis	Ethyl acetate fraction of *Amomum villosum* var. *xanthioides* enhances antioxidant capacity and improves oxidative status by increasing MT1 expression	[91]
